# Mutation Profiling of Premalignant Colorectal Neoplasia

**DOI:** 10.1155/2019/2542640

**Published:** 2019-11-12

**Authors:** Jakub Karczmarski, Krzysztof Goryca, Jacek Pachlewski, Michalina Dabrowska, Kazimiera Pysniak, Agnieszka Paziewska, Maria Kulecka, Malgorzata Lenarcik, Andrzej Mroz, Michal Mikula, Jerzy Ostrowski

**Affiliations:** ^1^Department of Genetics, Maria Sklodowska-Curie Institute-Oncology Center, 02-781 Warsaw, Poland; ^2^Department of Gastroenterology, Hepatology and Clinical Oncology, Center for Postgraduate Medical Education, 01-813 Warsaw, Poland; ^3^Department of Pathology and Laboratory Diagnostics, Maria Sklodowska-Curie Institute-Oncology Center, 02-781 Warsaw, Poland; ^4^Department of Pathomorphology, Center for Postgraduate Medical Education, 01-813 Warsaw, Poland

## Abstract

Accumulation of allelic variants in genes that regulate cellular proliferation, differentiation, and apoptosis may result in expansion of the aberrant intestinal epithelium, generating adenomas. Herein, we compared the mutation profiles of conventional colorectal adenomas (CNADs) across stages of progression towards early carcinoma. DNA was isolated from 17 invasive adenocarcinomas (ACs) and 58 large CNADs, including 19 with low-grade dysplasia (LGD), 21 with LGD adjacent to areas of high-grade dysplasia and/or carcinoma (LGD-H), and 28 with high-grade dysplasia (HGD). Ion AmpliSeq Comprehensive Cancer Panel libraries were prepared and sequenced on the Ion Proton. We identified 956 unique allelic variants; of these, 499 were considered nonsynonymous variants. Eleven genes (*APC*, *KRAS*, *SYNE1*, *NOTCH4*, *BLNK*, *FBXW7*, *GNAS*, *KMT2D*, *TAF1L*, *TCF7L2*, and *TP53*) were mutated in at least 15% of all samples. Out of frequently mutated genes, *TP53* and *BCL2* had a consistent trend in mutation prevalence towards malignancy, while two other genes (*HNF1A* and *FBXW7*) exhibited the opposite trend. HGD adenomas had significantly higher mutation rates than LGD adenomas, while LGD-H adenomas exhibited mutation frequencies similar to those of LGD adenomas. A significant increase in copy number variant frequency was observed from LGD through HGD to malignant samples. The profiling of advanced CNADs demonstrated variations in mutation patterns among colorectal premalignancies. Only limited numbers of genes were repeatedly mutated while the majority were altered in single cases. Most genetic alterations in adenomas can be considered early contributors to colorectal carcinogenesis.

## 1. Introduction

Cancers are highly heterogeneous, polygenic disorders that arise in multistep microevolutionary processes involving the selection of successive cellular clones that occur in response to specific environmental factors, as well as genetic influences. Colorectal cancer (CRC) is a common epithelial neoplasia worldwide and a leading cause of cancer-related morbidity and mortality [1]. As a result of the selective growth advantage of dysplastic cells over their normal neighbors, the morphological consequences of molecular alterations lead to progressive cytological and architectural disorganization, recognizable as the adenoma-carcinoma sequence, first described by Fearon and Vogelstein [2]. There are multiple colorectal neoplastic pathways, including the chromosomal instability (CIN) pathway, the microsatellite instability (MSI) pathway, and the CpG island methylator pathway (CIMP, also referred to as the serrated neoplasia pathway) [3].

Most CRCs are sporadic, with only 5–10% tumors developing as part of highly penetrant hereditary syndromes, mediated by rare germline mutations in genes involved in DNA mismatch repair or the adenomatous polyposis coli (*APC*) gene [4]. According to a study by The Cancer Genome Atlas (TCGA) of approximately three-quarter CRCs associated with MSI, around 15% exhibit hypermutation [5]. Nonhypermutated tumors carry common “driver” mutations in *APC*, *TP53*, *KRAS*, *PIK3CA*, *FBXW7*, *SMAD4*, *TCF7L2*, *NRAS*, *CTNNB1*, *SMAD2*, *FAM123B*, *SOX9*, *ATM*, and *ARID1A*, while hypermutated tumors commonly have alterations in *ACVR2A*, *APC*, *TGFBR2*, *BRAF*, *MSH3*, *MSH6*, *SLC9A9*, and *TCF7L2*. In both nonhypermutated and hypermutated tumors, alterations in “cancer genes” target the WNT, RTK/RAS, PI3K, TGF-*β*, and TP53 pathways [6], demonstrating that the genetic complexity of CRC is likely limited to various mutations within signaling and metabolic pathways [3]. At the chromosomal level, nonhypermutated tumors tend to be aneuploid, while hypermutated tumors are near-diploid [6].

Histologically, conventional colorectal adenomas (CNADs) are classified based on their proportions of villous components (tubular, tubulovillous, or villous adenoma) and the severity of dysplasia (low grade or high grade) [7]. Villous growth and high-grade dysplasia (HGD) are the most important determinants associated with the risk of adenomas transforming into malignant growths and are also closely related to adenoma size [8]; however, most adenomas stabilize their growth progression or even regress [9]. Mutations of *APC*, *KRAS*, and *β-catenin* represent key events in the development of adenomas, while mutations of *PIK3CA* and *TP53* occur during progression to invasive CRC [10–12]. The earliest clinically relevant CRCs are tumors that invade the submucosa, but not the muscular layer. It remains unknown which molecular alterations induce the final transition towards invasive growth; therefore, to prevent CRC, both adenomas and premalignant serrated polyps should be resected [3].

While large-scale sequence profiling of CRCs has advanced the understanding of their genetic characteristics, our knowledge of benign and premalignant CNADs remains limited. Recently determined mutation profiles, comprising limited numbers of genes, can clearly distinguish CNADs and CRCs [13, 14]. In this study, we compared the mutation profiles and abundance during progression of CNADs towards early carcinoma by deep sequencing the coding regions of 409 “cancer genes.” Consistent with previous reports [5, 13, 14], total numbers of nonsynonymous variants were significantly higher in adenomas exhibiting HGD than in those with low-grade dysplasia (benign adenomas); however, they did not differ between benign adenomas and carcinomas.

## 2. Material and Methods

This was a retrospective study using a collection of paraffin-embedded colorectal polyps removed by endoscopic polypectomy at the Maria Sklodowska-Curie Memorial Cancer Center and Institute of Oncology, Warsaw, Poland, between 2010 and 2016. Based on pathology reports, 58 adenomas ≥ 2 cm were reevaluated by referral pathologists (second opinion), 18 polyps were excised in one piece, and 40 were removed using a piecemeal technique. Patient characteristics are detailed in Table 1.

### 2.1. Compliance with Ethical Standards

All procedures were performed in accordance with the ethical standards of the local bioethical committee who gave permission for this retrospective study (approval ID: 13/2008 and 3/2019) and according to the principles of the 1964 Declaration of Helsinki.

### 2.2. DNA Isolation and Sequencing Using the Ion AmpliSeq Comprehensive Cancer Panel

Several series of sections were prepared from different parts of each specimen, and the upper and lower sections from each group were evaluated by pathologists to control for the relative cell type content. DNA was isolated from sections representative of a given component (tubular or villous adenoma) that contained the highest percentages of epithelial cells. In addition, fragments of polyps containing HGD and/or a carcinoma invading the submucosa were macrodissected. In total, DNA was extracted from 85 tumor samples using a QIAamp DNA FFPE (formalin-fixed paraffin-embedded) Tissue Kit (Qiagen), following the manufacturer's protocol.

DNA sample concentrations were measured using a Qubit fluorometer (Invitrogen), following the manufacturer's instructions, and stored at -20°C. Ion AmpliSeq Comprehensive Cancer Panel libraries were prepared from DNA for analysis of the coding regions of 409 oncogenes and tumor suppressor genes by sequencing using the Ion Proton system (Thermo).

### 2.3. Postsequencing Data Analyses and Variant Calling

Raw sequence reads were processed using the Torrent Suite analysis pipeline and mapped to the human genome assembly hg19 using TMAP. Variant calls were made with Torrent Variant Caller (version 5.6.0), using default parameters for somatic variants. Called variants were filtered with bcftools (version 1.3) using the following parameters: phred-scaled genotype quality (GQ) ≥ 5, read depth (DP) ≥ 20, flow evaluator alternate allele observation count (FAO) ≥ 4 for indels and ≥2 for SNPs, flow evaluator read depth (FDP) > 6 for SNPs and >10 for indels, strand bias in a variant relative to reference (STB) ≤ 0.9 for SNPs, and number of consecutive repeats of the alternate allele in the reference genome (HRUN) ≤ 6 for indels. Filtered variants were further filtered using the fpfilter tool with default parameters except for the following: --min-strandedness, 0.05; --max-mapqual-diff, 10; --max-readlen-diff, 10; and --max-mm-qualsum-diff, 50. Variants with alternate allele observations < 20% of total allele observations were discarded. Annotation of variants and prediction of their consequences for mature proteins were conducted using ANNOVAR [15], while deleteriousness was assessed using the SIFT [16] and PolyPhen [17] algorithms. Variants with population frequencies > 0.001, according to the 1000 Genomes Project database (European and global), the Exome Sequencing Project of the National Heart, Lung, and Blood Institute [18], and the Exome Aggregation Consortium database [19], were discarded. To reduce the probability of listing a germline variant specific for the local population, we removed all variants present in more than 35% of samples.

Copy number variations (CNVs) were called using Contra (version 2.0.8, [20]) with default parameters (except –minReadDepth, which was set to 32), and a reference baseline was created using sequencing results from 78 blood samples collected from patients with pancreatic cysts analyzed in parallel for another project. CNVs called in ≥20% of samples were discarded as possible false-positive results.

### 2.4. Driver Mutation and Nonsynonymous Variant Analysis

Two types of mutation were specified: nonsynonymous rare mutations and the so-called “driver” mutations. Nonsynonymous variants were considered driver mutations if they fulfilled at least one of the following conditions: (i) a known driver gene (as designated by CRAVAT [21]), (ii) cancer driver FDR of ≤0.1 (computed using CHASM [22]), and (iii) a gene with a cancer driver FDR value of ≤0.1.

The trend for changes in mutation frequencies in benign polyp samples through to those in malignant specimens was assessed using the Cochran-Armitage trend test. Changes in the numbers of driver and nonsynonymous mutations were assessed by linear regression, with benign polyps as the reference.

A random forest classifier was prepared for three groups, benign adenomas, HG adenomas, and carcinomas, taking into account the presence or absence of nonsynonymous and driver mutations in genes. The significance of *p* values was assessed using the rfPermute package (https://cran.r-project.org/web/packages/rfPermute/index.html, Eric Archer).

## 3. Results

To establish genetic profiling across a spectrum of colorectal neoplasias, we sequenced 409 cancer-related gene coding regions in 85 samples dissected from 58 CNADs > 2 cm. Among these, 19 samples were from adenomas (ten tubular and nine tubulovillous) containing only low-grade dysplasia (LGD, benign adenomas), 21 were from adenomas with LGD associated with synchronous high-grade dysplasia adenoma and/or carcinoma components (premalignant-related adenomas) (LGD-H), 28 were from high-grade dysplasia (HGD) adenomas, and 17 were from submucosal invasive adenocarcinoma (AC). For the purpose of statistical analysis of mutation frequencies, LGD and LGD-H groups were treated as one.

**Figure 1:**
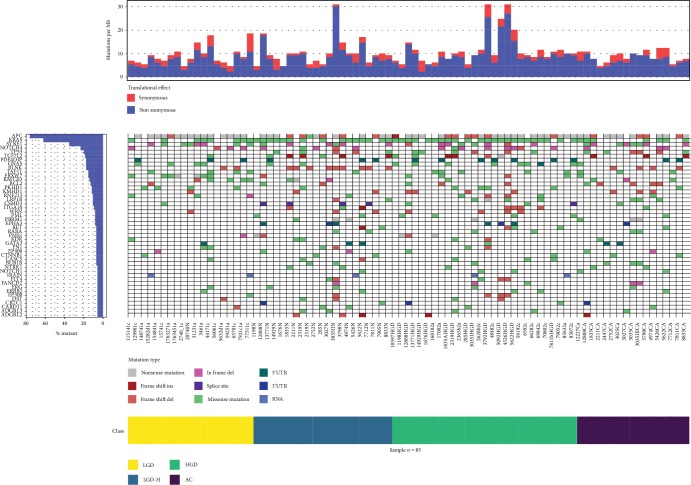
The structure of genetic variance among the investigated samples. Waterfall plot of genes mutated in >5% of samples. Mutation frequency, shown in the top panel, is calculated relative to the assayed DNA length (1.29 Mb). LGD: low-grade dysplasia; HGD: high-grade dysplasia; LGD-H: low-grade dysplasia adjacent to areas of high-grade dysplasia and/or carcinoma; AC: adenocarcinoma.

The median sequencing depth was 750×, and >95% of targeted sequences were covered at ≥50× in 76 samples (90%). We identified 956 unique single-nucleotide variants across the 409 genes included in the Comprehensive Cancer Panel ([Supplementary-material supplementary-material-1]). Among them, 499 were considered nonsynonymous allelic variants. The average genetic variant rate was 15.7/Mb, and the nonsynonymous variant rate was 8.0/Mb; the mean number of nonsynonymous variants discovered per sample was 10.3. In total, 1632 point mutations were detected in sequences included in the Comprehensive Cancer Panel. The average rate of mutations in The Cancer Genome Atlas (TCGA) CRC dataset was 8.8/Mb. While in five (5.9%) samples, nonsilent mutation rates were 20–30/Mb, none could be considered hypermutated. The structure of genetic variance among the investigated samples is presented in Figure 1.

Eight hundred and forty-three unique CNVs were identified across 292 genes. The mean number of CNVs in LGD samples (12.4) was significantly lower than that in HGD samples (43.6, *p* = 0.0004). Similarly, the mean number of CNVs in CA samples (76.1) was significantly higher than that in HGD samples (*p* = 0.027).

Nonsynonymous variants ([Supplementary-material supplementary-material-1]) were submitted to further analysis. In total, 92 driver genes were selected ([Supplementary-material supplementary-material-1]). Nine genes were consistently mutated; i.e., they carried a nonsynonymous mutation in at least one sample per group and were mutated in ≥15% of samples on average (Table 2(a)), and two other genes were mutated in ≥15% of samples on average but not mutated in one of the tumor groups (Table 2(b)).

Six genes demonstrated a statistically significant trend in changes for nonsynonymous variant frequencies from nonmalignant to malignant groups (nominal *p* value < 0.05), of which *HNF1A*, *TP53*, *FBXW7*, and *BCL2* were mutated in ≥10% of samples on average. The mutation proportions for *TP53* and *BCL2* rose, while those of *FBXW7* and *HNF1A* declined along progression to CA. These four genes also exhibited a significant trend in the proportion of driver mutations since all nonsynonymous mutations were considered driver mutations (Tables 2(a) and 2(b), [Supplementary-material supplementary-material-1]).

A random forest classifier designated 10 genes, in which the presence or absence of a driver mutation was statistically significant in the classification of at least one group (nominal *p* < 0.05, Table 3). Three of these genes were also important for the construction of the entire model: *KRAS*, *HNF1A*, and *TP53.*

## 4. Discussion

Stepwise, nonrandom accumulation of allelic variants in genes that regulate cellular proliferation, differentiation, and apoptosis can cause expansion of the aberrant intestinal epithelium into adenomas. Sequence alterations in specific genes, including *APC* and *KRAS*, contribute to the development of early polypoid lesions, while other genetic aberrations, such as inactivating mutations of *TP53*, can promote malignancy and are observed in more advanced stages of CRC development [23]. Intratumor heterogeneity, resulting from the presence of different subclones, can lead to discordance in the mutation landscapes of tumor cells isolated from different components of adenomatous polyps. For example, 43% of adenoma components and 51% of carcinoma components from 70 tumor samples comprising both adenoma and carcinoma were positive for *KRAS* mutations, while 23% generated discordant results [24]. Furthermore, *KRAS* mutations were identified in a small number of samples from the histologically normal colonic mucosa adjacent to carcinomas [25].

Mutations that provide a growth advantage are driver alterations, while those that occur coincidentally alongside drivers are referred to as passenger events [26]. Allelic variants in adenomas can be considered early driver events that contribute to the initiation of tumorigenesis, while those enriched in carcinomas can be classified as later driver mutations involved in tumor progression. Despite the high mutation loads of adenomas, the contributions of various mutations to oncogenesis differ [27]. Consequently, only a small percentage of adenomas will progress to become CRC, with the majority remaining stable over time or even regressing. While <5% of small tubular adenomas with LGD will transform into CRC [28], the 10-year cumulative risk of an advanced adenoma (≥25% villous component, HGD, or size ≥ 10 mm) developing into cancer is 25% for patients aged 55 years and increases to approximately 40% for those aged 80 years [29]; thus, advanced adenomas have a higher malignancy potential than nonadvanced adenomas [3]. Nevertheless, it remains unknown which molecular alterations induce the final transition towards malignancy.

We analyzed mutation profiles in components of CNADs comprised entirely of either LGD or LGD with synchronous HGD and/or carcinoma components. As corresponding normal samples were unavailable, we employed highly stringent filtering, based on existing databases of variants in the general population. All variants present in >0.1% of populations were discarded, resulting in a >10-fold reduction in the variant dataset. Using the Ion AmpliSeq Comprehensive Cancer Panel, which provides multiplexed targeted selection of all exons of 409 tumor suppressor genes and oncogenes implicated in cancer, we identified 956 unique variants (after all filtering steps), of which 499 were considered nonsynonymous allelic variants in 214 “cancer genes.” Among these mutated genes, consistent with previous studies [5, 13, 14], *APC*, *KRAS*, and *SYNE1* were mutated in 76.5%, 62.3%, and 35.3% of samples, respectively, and another 11 genes (*BCL2*, *BLNK*, *FBXW7*, *GNAS*, *LRP1B*, *MLL2/KMT2D*, *MLL3/KMT2C*, *PKHD1*, *RNF213*, *TAF1L*, *TCF7L2*, and *TP53*) were mutated in ≥10% of all samples. While the majority of allelic variants were found in individual cases, all genes that were mutated in two or more carcinoma components were also altered at least in one adenoma component. “Private” variants likely arise at an early stage of adenoma development, generating multiclonal adenomas [30].

In a study of the mutation profiles of synchronous colon adenoma and CRC using whole exome sequencing (WES), Lee et al. [14] found nonsilent allelic variants in the cancer census genes, *APC*, *KRAS*, *TP53*, *GNAS*, *NRAS*, *SMAD4*, *ARID2*, and *PIK3CA*, in 12 HGD adenomas, which matched sequences in classical adenoma-carcinomas, and reported allelic variants in *MTOR*, *ACVR1B*, *GNAQ*, *ATM*, *CNOT1*, *EP300*, *ARID2*, *RET*, and *MAP2K4* in colon adenomas for the first time [2, 10]. Lin et al. discovered four additional affected genes (*CTNNB1*, *KRTAP4-5*, *GOLGA8B*, and *TMPRSS13*) in adenomas that represented potential new somatic driver mutations with characteristics of oncogenes [13]. The majority of mutated genes previously reported in adenomas [5, 13, 14] were also found in our study; however, most were present at a low frequency.

Of 138 potential driver genes (74 tumor suppressor genes and 64 oncogenes), a typical sporadic CRC may only contain 2–8 driver gene alterations, making each tumor unique [31–33]. By covering 9423 tumor exomes using 26 computational tools, 299 driver genes were cataloged recently in the contexts of their anatomical sites and cancer/cell types [34]. *APC*, *KRAS*, *BRAF*, *PIK3CA*, *SMAD4*, and *TP53* are the most common “drivers” among late CRCs [35]; however, distinguishing rare driver mutations from passengers remains challenging. Based on strict criteria for driver gene classification, we selected 92 affected genes in advanced adenomas that could be considered early drivers in colorectal tumorigenesis.

Genes displaying a consistent trend in mutation prevalence from nonadvanced to advanced adenomas and CRC could reflect progress towards malignancy [13]. While mutations in *TP53* and *PIK3CA* are characteristic of late-stage CRC [36], pathogenic *TP53* allelic variants have also been reported in colon adenomas [10, 14]. In this study, 0%, 21.4%, and 35.3% of LG adenomas, HG adenomas, and synchronous carcinoma components, respectively, were affected by *TP53* mutations. *TP53* was one of the two genes with a significant consistent trend in mutation prevalence towards malignancy, while four other genes (*HNF1A*, *KAT6B*, *FBXW7*, and *NFKB1*) exhibited the opposite trend, with mutation frequencies decreasing towards carcinoma. Similar increases followed by decreases in the frequency of a particular gene mutation during colon tumorigenesis have been noted previously [37]. These may represent mutations acting as drivers during a specific phase of colorectal carcinogenesis, followed by loss when their role is no longer essential for growth advantage [37, 38]. It is also possible that the observed decreased or increased frequencies in mutation profiles result from different levels of development towards malignancy across the adenoma tissue.

Genes involved in WNT signaling are highly conserved through evolution, and recurrent mutations in multiple genes encoding key proteins in the canonical WNT/*β*-catenin signaling pathway occur in a wide spectrum of human cancers [39, 40]. Among these genes, *APC* and *CTNNB1* (which encodes *β*-catenin) are key factors in the reprogramming of the nuclear T cell factor/lymphoid enhancer factor (TCF/LEF) transcriptional network. The majority of *APC* mutations in colorectal neoplasia are truncating and affect the WNT pathway [35]. As expected, we found that *APC* was the most frequently mutated gene across the adenomas studied, while other WNT pathway genes (*CTNNB1*, *EP300*, *TCF7L2*, and *AMER1*) were altered less frequently. All of these genes are implicated as drivers of WNT-dependent tumor growth [39]. Both *APC* and *CTNNB1* are classic oncogenes, while *AMER1* (also known as the Wilms tumor gene on the X chromosome, *WTX*) is a tumor suppressor gene [41]. In addition to WNT-related genes, other mutated genes, including *BCL2*, *FBXW7*, *GNAS*, *HNF1A*, *KRAS*, *MLL2/KMT2D*, *MLL3/KMT2C*, *SYNE1*, *TCF7L2*, *TP53*, *NOTCH1*, *PBRM1*, *RET*, *RARA*, and *FN1*, can be considered drivers of colorectal tumorigenesis.

Structural variations in the human genome, including deletions, insertions, duplications, and large-scale copy number variants, are collectively termed CNVs. CNVs are influential factors in gene expression in both normal and various cancer tissues [42–44]. As expected, we detected a progressive increase in CNVs from LGD through HGD to malignant samples. Most sporadic CRCs are CIN and may be partly attributable to somatic *APC* mutations [45]. However, the first large-scale genome-wide analysis investigating rare CNVs in sporadic CRC indicated that rare CNVs increased the risk of CRC and that the assembly of chromatin or nucleosome-related or olfaction-associated genes might contribute to this elevated risk [46]. Thus, the genomic instability noted in CRC tumorigenesis may not be associated with *APC* mutations or its associated alterations within the WNT signaling pathway. Interestingly, comparisons of genetic aberrations detected in normal colon samples from patients with CRC with those found in corresponding polyp tissue and peripheral blood indicate that CNVs were present, not only in tumor tissues but also in the normal colon and blood [47].

In summary, we confirmed an increase in the mutation load in HGD compared with LGD adenomas, while carcinoma components of adenomas had mutation loads similar to those of LGD adenomas (Figure 1). Furthermore, the number of CNVs progressively increased in samples from adenomas representing LGD, HGD, and CA samples. Most genetic alterations detected in this study, including those involved in the WNT signaling pathway, can be considered early contributors to colorectal carcinogenesis [48]; however, only a limited number of genes were consistently mutated in ≥10% of cases, while most gene changes affected single cases. The ultimate contribution of these mutations to the process of CRC tumorigenesis remains unclear.

## Figures and Tables

**Table 1:** Clinicopathologic parameters of 58 patients with colorectal adenoma.

No.	Age	Sex	Diameter (cm)	Location in colon	Microscopic evaluation	Dysplasia	Accompanied by a malignant lesion
1	63	M	2	Descending	Tubular adenoma	LGD	No
2	76	F	6	Sigmoid	Tubular adenoma	LGD	No
3	71	M	2	Sigmoid	Tubular adenoma	LGD	No
4	69	F	2	Sigmoid	Tubular adenoma	LGD	No
5	65	F	2	Sigmoid	Tubular adenoma	LGD	No
6	77	M	4.5	Descending	Tubular adenoma	LGD	No
7	65	F	3	Sigmoid	Tubular adenoma	LGD	No
8	86	M	3.5	Splenic	Tubular adenoma	LGD	No
9	63	F	4	Ascending	Tubular adenoma	LGD	No
10	77	F	2	Sigmoid	Tubular adenoma	LGD	No
11	54	F	2	Ascending	Tubular adenoma	HGD	No
12	42	F	2.6	Sigmoid	Tubular adenoma	HGD	No
13	70	M	2	Cecum	Tubular adenoma	HGD	Yes
14	64	F	4	Ascending	Tubular adenoma	HGD	Yes
15	63	F	3	Sigmoid	Tubular adenoma	HGD	Yes
16	58	M	3	Cecum	Tubular adenoma	HGD	Yes
17	69	F	2	Sigmoid	Tubulovillous adenoma	LGD	No
18	66	F	2	Descending	Tubulovillous adenoma	LGD	No
19	31	F	3	Sigmoid	Tubulovillous adenoma	LGD	No
20	72	M	3	Rectum	Tubulovillous adenoma	LGD	No
21	78	M	2	Rectum	Tubulovillous adenoma	LGD	No
22	60	M	3	Descending	Tubulovillous adenoma	LGD	No
23	56	M	3.5	Cecum	Tubulovillous adenoma	LGD	No
24	79	F	3.5	Sigmoid	Tubulovillous adenoma	LGD	No
25	54	F	4	Sigmoid	Tubulovillous adenoma	LGD	No
26	76	M	3	Sigmoid	Tubulovillous adenoma	LGD	No
27	74	M	3.5	Rectum	Tubulovillous adenoma	HGD	No
28	54	M	4	Sigmoid	Tubulovillous adenoma	HGD	No
29	55	F	3	Rectum	Tubulovillous adenoma	HGD	No
30	57	M	2	Cecum	Tubulovillous adenoma	HGD	No
31	80	F	3	Rectum	Tubulovillous adenoma	HGD	No
32	71	F	6	Rectum	Tubulovillous adenoma	HGD	No
33	73	F	4	Sigmoid	Tubulovillous adenoma	HGD	No
34	69	M	6	Rectum	Tubulovillous adenoma	HGD	No
35	64	M	3	Rectum	Tubulovillous adenoma	HGD	No
36	57	M	6	Rectum	Tubulovillous adenoma	HGD	No
37	58	M	4	Splenic	Tubulovillous adenoma	HGD	No
38	70	F	3.5	Rectum	Tubulovillous adenoma	HGD	No
39	63	M	3.5	Transverse	Tubulovillous adenoma	HGD	No
40	78	F	4	Rectum	Tubulovillous adenoma	HGD	No
41	63	M	4	Rectum	Tubulovillous adenoma	HGD	No
42	57	F	3.5	Sigmoid	Tubulovillous adenoma	HGD	No
43	64	F	3	Transverse	Tubulovillous adenoma	HGD	No
44	72	M	5	Rectum	Tubulovillous adenoma	HGD	No
45	58	M	3.5	Descending	Tubulovillous adenoma	HGD	No
46	66	F	6	Rectum	Tubulovillous adenoma	HGD	Yes
47	80	F	6	Rectum	Tubulovillous adenoma	HGD	Yes
48	55	F	3	Sigmoid	Tubulovillous adenoma	HGD	Yes
49	73	M	3.5	Rectum	Tubulovillous adenoma	HGD	Yes
50	53	F	4	Rectum	Tubulovillous adenoma	HGD	Yes
51	68	M	3	Rectum	Tubulovillous adenoma	HGD	Yes
52	70	M	4	Sigmoid	Tubulovillous adenoma	HGD	Yes
53	90	M	3.5	Rectum	Tubulovillous adenoma	HGD	Yes
54	71	M	4	Descending	Tubulovillous adenoma	HGD	Yes
55	65	M	5	Rectum	Tubulovillous adenoma	HGD	Yes
56	53	M	4.5	Rectum	Tubulovillous adenoma	HGD	Yes
57	76	F	6	Rectum	Tubulovillous adenoma	HGD	Yes
58	93	M	5	Rectum	Tubulovillous adenoma	HGD	Yes

LGD: low-grade dysplasia; HGD: high-grade dysplasia.

**(a):** 

Gene	LGD, *n* (%)	LGD-H, *n* (%)	HGD, *n* (%)	AC, *n* (%)	Mean (%)	*p* value
*APC*	15 (78.9)	14 (66.7)	23 (82.1)	13 (76.5)	76.5	0.785
*KRAS*	8 (42.1)	15 (71.4)	23 (82.1)	7 (41.1)	62.4	0.640
*SYNE1*	8 (42.1)	6 (28.6)	8 (28.6)	8 (47.1)	35.3	0.994
*NOTCH4*	4 (21.1)	5 (23.8)	8 (28.6)	3 (17.6)	23.5	0.858
*TCF7L2*	1 (5.3)	6 (28.6)	6 (21.4)	3 (17.6)	18.8	0.441
*GNAS*	4 (21.1)	2 (9.5)	8 (28.6)	1 (5.9)	17.6	0.561
*FBXW7*	7 (36.8)	1 (4.8)	4 (14.3)	1 (5.9)	15.3	0.029
*TAF1L*	4 (21.1)	1 (4.8)	5 (17.9)	3 (17.6)	15.3	0.903
*MLL2/KMT2D*	5 (26.3)	3 (14.3)	1 (3.6)	3 (17.6)	15.33	0.226
*BCL2*	1 (5.3)	2 (9.5)	3 (10.7)	5 (29.4)	12.9	0.047
*MLL3/KMT2C*	1 (5.3)	2 (9.5)	4 (14.3)	3 (17.6)	11.8	0.205
*PKHD1*	4 (21.1)	1 (4.8)	4 (14.3)	1 (5.9)	11.8	0.326
*RNF213*	3 (15.8)	1 (4.8)	4 (14.3)	1 (5.9)	10.6	0.601
*CSMD3*	1 (5.3)	4 (19.0)	3 (10.7)	1 (5.9)	10.6	0.836

**(b):** 

Gene	LGD, *n* (%)	LGD-H, *n* (%)	HGD, *n* (%)	AC, *n* (%)	Mean (%)	*p* value
*TP53*	0	5 (23.8)	6 (21.4)	6 (35.3)	20.0	0.015
*BLNK*	2 (10.5)	9 (42.9)	0	4 (23.5)	17.6	0.666
*HNF1A*	7 (36.8)	2 (9.5)	2 (7.1)	0	12.9	0.001
*LRP1B*	0	2 (9.5)	4 (14.3)	3 (17.6)	10.6	0.067

LGD: low-grade dysplasia; HGD: high-grade dysplasia; LGD-H: low-grade dysplasia adjacent to areas of high-grade dysplasia and/or carcinoma; AC: adenocarcinoma.

**Table 3:** Random forest classifier results for different groups with a decrease in accuracy for each group, *p* values computed by the rfPermute package, and a mean decrease in accuracy for overall classification.

Gene	LGD: decrease in accuracy	LGD: *p* value	HGD: decrease in accuracy	HGD: *p* value	AC: decrease in accuracy	AC: *p* value	Mean decrease in accuracy	Mean decrease in accuracy: *p* value
*KRAS*	4.320	0.050	15.685	0.010	2.391	0.139	13.029	0.010
*HNF1A*	8.091	0.030	9.131	0.020	5.828	0.030	11.724	0.020
*TP53*	7.991	0.030	-2.460	0.772	2.618	0.109	5.094	0.069
*FBXW7*	2.640	0.168	8.457	0.030	4.272	0.050	8.780	0.040
*TCF7L2*	4.941	0.050	4.758	0.050	2.637	0.149	6.294	0.040
*MLL2/KMT2D*	1.191	0.238	7.749	0.030	-0.198	0.426	5.456	0.059
*BCL2*	0.359	0.337	5.242	0.059	3.657	0.109	6.076	0.030
*RARA*	1.728	0.188	2.823	0.337	5.263	0.050	5.458	0.109
*SYNE*	-0.621	0.535	6.495	0.040	-2.310	0.812	3.814	0.178

LGD: low-grade dysplasia; HGD: high-grade dysplasia; AC: adenocarcinoma.

## Data Availability

The sequencing datasets are not publicly available due to a concern to protect individual patient confidentiality but are available from the corresponding author on reasonable request.

## Supplementary Materials

Supplementary Materials Supplementary Table S1: variants identified in assessed samples: Gene—symbol of a gene the given variant maps or the names of the nearest genes; Func.refGene—location of the variant relative to the gene; census—indication if a given gene is associated with CRC development according to Gene Cancer Census; Chromosome, position—genome coordinates of the variant; REF/ALT—reference/alternative variant sequence; variantsFound—number of samples given variant was found; GT—genotype detected in a given sample (0: reference, 1: alternative); SAF/SAR—number of reads on forward/reverse strand supporting alternative variant; DP/SAF/SAR—number of high quality reads/nonreference reads from the forward strand/nonreference reads from the reverse strands, respectively, at a given position in a given patient; ExAC_XXX—frequency of the alternative variant in XXX population according to the ExAC database; 1000g_all/eur—variant frequency in the 1000 Genomes Project database (total/European); esp6500siv2_all—variant frequency according to National Heart, Lung, and Blood Institute GO Exome Sequencing Project; SIFT/Polyphen2/LRT/FATHMM/RadialSVM “_pred”—prediction of variant impact on protein structure: B—benign, N—neutral, T—tolerated, and D—deleterious; and ICGC_Id—variant ID in the ICGC database (known cancer-related variants). Heterozygous variants are marked orange, homozygous are marked red. Supplementary Table 2: driver genes and criteria for inclusion: TSG—tumor suppressor gene, Oncogene; CHASM—gene included for its composite *p* value in the CHASM; CHASM-single—gene with single driver mutations, according to CHASM. Supplementary Table 3: full results for Cochran-Armitage trend test for nonsynonymous and driver mutations.

## References

[1] Gaj P., Maryan N., Hennig E. E. (2012). Pooled sample-based GWAS: a cost-effective alternative for identifying colorectal and prostate cancer risk variants in the Polish population. *PLoS One*.

[2] Fearon E. R., Vogelstein B. (1990). A genetic model for colorectal tumorigenesis. *Cell*.

[3] IJspeert J. E. G., Medema J. P., Dekker E. (2015). Colorectal neoplasia pathways: state of the art. *Gastrointestinal Endoscopy Clinics of North America*.

[4] Vogelstein B., Fearon E. R., Hamilton S. R. (1988). Genetic alterations during colorectal-tumor development. *The New England Journal of Medicine*.

[5] The Cancer Genome Atlas Network (2012). Comprehensive molecular characterization of human colon and rectal cancer. *Nature*.

[6] Cheasley D., Jorissen R. N., Liu S. (2015). Genomic approach to translational studies in colorectal cancer. *Translational Cancer Research*.

[7] Rubio C. A., Nesi G., Messerini L. (2006). The Vienna classification applied to colorectal adenomas. *Journal of Gastroenterology and Hepatology*.

[8] Atkin W., Valori R., Kuipers E. (2012). European guidelines for quality assurance in colorectal cancer screening and diagnosis. First Edition--Colonoscopic surveillance following adenoma removal. *Endoscopy*.

[9] Risio M. (2012). The natural history of colorectal adenomas and early cancer. *Der Pathologe*.

[10] Baker S. J., Fearon E. R., Nigro J. M. (1989). Chromosome 17 deletions and p53 gene mutations in colorectal carcinomas. *Science*.

[11] Thiagalingam S., Lengauer C., Leach F. S. (1996). Evaluation of candidate tumour suppressor genes on chromosome 18 in colorectal cancers. *Nature Genetics*.

[12] Parsons D. W., Wang T.-L., Samuels Y. (2005). Mutations in a signalling pathway. *Nature*.

[13] Lin S.-H., Raju G. S., Huff C. (2018). The somatic mutation landscape of premalignant colorectal adenoma. *Gut*.

[14] Lee S. H., Jung S. H., Kim T.-M. (2017). Whole-exome sequencing identified mutational profiles of high-grade colon adenomas. *Oncotarget*.

[15] Wang K., Li M., Hakonarson H. (2010). ANNOVAR: functional annotation of genetic variants from high-throughput sequencing data. *Nucleic Acids Research*.

[16] Ng P. C., Henikoff S. (2003). SIFT: predicting amino acid changes that affect protein function. *Nucleic Acids Research*.

[17] Adzhubei I., Jordan D. M., Sunyaev S. R. (2013). Predicting Functional Effect of Human Missense Mutations Using PolyPhen‐2. *Current Protocols in Human Genetics*.

[18] Sudmant P. H., Rausch T., Gardner E. J. (2015). An integrated map of structural variation in 2,504 human genomes. *Nature*.

[19] Lek M., Karczewski K. J., Minikel E. V. (2016). Analysis of protein-coding genetic variation in 60,706 humans. *Nature*.

[20] Li J., Lupat R., Amarasinghe K. C. (2012). CONTRA: copy number analysis for targeted resequencing. *Bioinformatics*.

[21] Douville C., Carter H., Kim R. (2013). CRAVAT: cancer-related analysis of variants toolkit. *Bioinformatics*.

[22] Carter H., Chen S., Isik L. (2009). Cancer-specific high-throughput annotation of somatic mutations: computational prediction of driver missense mutations. *Cancer Research*.

[23] Punt C. J. A., Koopman M., Vermeulen L. (2017). From tumour heterogeneity to advances in precision treatment of colorectal cancer. *Nature Reviews. Clinical Oncology*.

[24] Hershkovitz D., Simon E., Bick T. (2014). Adenoma and carcinoma components in colonic tumors show discordance for KRAS mutation. *Human Pathology*.

[25] Burmer G. C., Loeb L. A. (1989). Mutations in the KRAS2 oncogene during progressive stages of human colon carcinoma. *Proceedings of the National Academy of Sciences of the United States of America*.

[26] Fimereli D., Fumagalli D., Brown D. (2018). Genomic hotspots but few recurrent fusion genes in breast cancer. *Genes, Chromosomes & Cancer*.

[27] Vaqué J. P., Martínez N., Varela I. (2015). Colorectal adenomas contain multiple somatic mutations that do not coincide with synchronous adenocarcinoma specimens. *PLoS One*.

[28] Muto T., Bussey H. J., Morson B. C. (1975). The evolution of cancer of the colon and rectum. *Cancer*.

[29] Brenner H., Hoffmeister M., Stegmaier C., Brenner G., Altenhofen L., Haug U. (2007). Risk of progression of advanced adenomas to colorectal cancer by age and sex: estimates based on 840,149 screening colonoscopies. *Gut*.

[30] Sottoriva A., Kang H., Ma Z. (2015). A Big Bang model of human colorectal tumor growth. *Nature Genetics*.

[31] Carethers J. M., Jung B. H. (2015). Genetics and genetic biomarkers in sporadic colorectal cancer. *Gastroenterology*.

[32] Vogelstein B., Papadopoulos N., Velculescu V. E., Zhou S., Diaz L. A., Kinzler K. W. (2013). Cancer genome landscapes. *Science*.

[33] Tomasetti C., Marchionni L., Nowak M. A., Parmigiani G., Vogelstein B. (2015). Only three driver gene mutations are required for the development of lung and colorectal cancers. *Proceedings of the National Academy of Sciences of the United States of America*.

[34] Bailey M. H., Tokheim C., Porta-Pardo E. (2018). Comprehensive characterization of cancer driver genes and mutations. *Cell*.

[35] Huang D., Sun W., Zhou Y. (2018). Mutations of key driver genes in colorectal cancer progression and metastasis. *Cancer Metastasis Reviews*.

[36] Fearon E. R. (2011). Molecular genetics of colorectal cancer. *Annual Review of Pathology*.

[37] Zauber P., Marotta S. P., Sabbath-Solitare M. (2016). GNAS gene mutation may be present only transiently during colorectal tumorigenesis. *International Journal of Molecular Epidemiology and Genetics*.

[38] Yamada M., Sekine S., Ogawa R. (2012). Frequent activating GNAS mutations in villous adenoma of the colorectum. *The Journal of Pathology*.

[39] Zhan T., Rindtorff N., Boutros M. (2017). Wnt signaling in cancer. *Oncogene*.

[40] Gao C., Wang Y., Broaddus R., Sun L., Xue F., Zhang W. (2018). Exon 3 mutations of CTNNB1 drive tumorigenesis: a review. *Oncotarget*.

[41] Huff V. (2011). Wilms’ tumours: about tumour suppressor genes, an oncogene and a chameleon gene. *Nature Reviews. Cancer*.

[42] Scherer S. W., Lee C., Birney E. (2007). Challenges and standards in integrating surveys of structural variation. *Nature Genetics*.

[43] Molparia B., Oliveira G., Wagner J. L., Spencer E. G., Torkamani A. (2018). A feasibility study of colorectal cancer diagnosis via circulating tumor DNA derived CNV detection. *PLoS One*.

[44] Liang L., Fang J.-Y., Xu J. (2016). Gastric cancer and gene copy number variation: emerging cancer drivers for targeted therapy. *Oncogene*.

[45] Rowan A. J., Lamlum H., Ilyas M. (2000). APC mutations in sporadic colorectal tumors: A mutational “hotspot” and interdependence of the “two hits”. *Proceedings of the National Academy of Sciences of the United States of America*.

[46] Li Z., Yu D., Gan M. (2015). A genome-wide assessment of rare copy number variants in colorectal cancer. *Oncotarget*.

[47] Druliner B. R., Ruan X., Sicotte H. (2018). Early genetic aberrations in patients with sporadic colorectal cancer. *Molecular Carcinogenesis*.

[48] Borras E., San Lucas F. A., Chang K. (2016). Genomic landscape of colorectal mucosa and adenomas. *Cancer Prevention Research (Philadelphia, Pa.)*.

